# Machine Learning and SHAP for Morphological Feature Identification of Longjing Tea Straightness

**DOI:** 10.3390/foods15213983

**Published:** 2026-11-06

**Authors:** Wenhan Fan, Siqi Zhang, Shuying Gong, Yingbin Zhang, Fangyuan Fan

**Affiliations:** 1 Tea Research Institute, College of Agriculture and Biotechnology, Zhejiang University, Hangzhou 310058, China; 2 Tea Research Institute, Chinese Academy of Agricultural Sciences, Hangzhou 310018, China

**Keywords:** Longjing tea, straightness, morphological features, machine learning, SHAP

## Abstract

The sensory evaluation of Longjing tea quality relies predominantly on subjective human judgment. Straightness is a critical sensory descriptor in the morphological quality assessment of Longjing tea, which lacks objective and quantifiable criteria. To objectively quantify this descriptor from a morphological perspective, this study developed an interpretable machine learning framework that integrates computer vision and SHAP analysis to digitally characterize the sensory attribute of straightness. Nine morphological features were extracted from Longjing tea samples collected across major production regions, including six contour-based and three skeleton-based descriptors. Three classification models, namely random forest (RF), extreme gradient boosting (XGBoost), and support vector machine (SVM), were constructed and compared based on morphological features. SHAP analysis was employed to interpret model decisions, identifying three core morphological determinants, length ratio (lr), standard deviation (sd) and rectangularity (R), which consistently ranked highest in both univariate effect sizes and cross-model feature importance, with Cliff’s δ of 0.93, 0.81 and −0.78, respectively. By establishing a quantitative link between morphological features and sensory terminology, this study offers an interpretable tool for automated tea quality evaluation, with broader implications for digital standardization in quality evaluation of agricultural products.

## 1. Introduction

Longjing tea, a traditional Chinese tea with a history spanning over 1200 years, is favored by consumers for its unique attributes “green color, fragrant aroma, mellow taste, and elegant appearance.” As an iconic agricultural product with geographical indication status, Longjing tea has gained widespread acceptance and consumption in China. Appearance is a critical determinant in Longjing tea quality assessment, affecting not only product recognizability but also brewing behavior. Furthermore, previous study indicated that leaves characterized by narrowness and a high length-to-width ratio exhibited quicker sinking during infusion [1]. Specifically, “straightness” is recognized as an important morphological feature within this framework. According to the Chinese National Standard “Tea vocabulary for sensory evaluation” (GB/T 14487-2017) [2], “straightness” is defined as the characteristic of tea leaves that are neither curved nor curled, yet it does not imply a perfectly straight line. However, what specific morphological attributes constitute this attribute remains undefined. Currently, the assessment of the straightness attribute relies solely on the subjective judgment of experienced panelists, lacking any quantitative standard, which often leads to considerable bias in practical evaluation.

Shape descriptors offer a quantitative framework for translating the appearance of agricultural products into measurable quality attributes. The most widely employed family of descriptors is derived from the object’s contour or its minimum bounding rectangle. For example, Wang et al. [3] utilized seven such contour descriptors (length, width, circularity, rectangularity, slightness, compactness and linearity) to monitor the moisture content of roasted green tea throughout roasting process. Notably, shape features were found to be more readily extracted by image processing than colour or texture. In fruit crops, where shape governs cultivar description, variety registration and market classification, supervised classifiers trained on forty-one morphological traits quantified by the Tomato Analyzer successfully reproduce four established tomato shape-grading systems with over 85% accuracy, outperforming expert visual assessment [4]. Such descriptors compress a contour into a few ratios, failing to represent asymmetric or locally bulged outline. Outline-wide methods overcome this limitation. For instance, elliptic Fourier descriptors (EFDs) decompose a closed contour into a sum of harmonically related ellipses, each harmonic contributing four coefficients, thus retaining the entire contour geometry rather than summarizing it by a single index [5]. Following normalisation of contour size and orientation, sixteen sets of EFD coefficients resolved six jujube cultivars across five developmental stages [5]. Additionally, combining EFDs with linear discriminant analysis separated morphologically similar grape cultivars that sphericity and elongation alone could not resolve [6]. This enhanced descriptive power, however, compromises comparability, as EFD coefficients are sensitive to planar translation, scaling and rotation and are further affected by the arbitrary choice of starting point and traversal direction. Direct comparison between different specimens is possible only after complete normalization, which further removes reversal and starting-point effects [7]. For a structure that is itself a curve, the analogous descriptor is a tortuosity index, which has been applied mainly to the vascular system, where the tortuosity of retinal, carotid and coronary vessels serves as a measure of vascular geometry and a marker of disease [8], but to our knowledge no application to the appearance of agricultural products has been reported. Overall, shape description has evolved from single geometric ratios to full-contour representations capable of automated classification. Yet, to date, these descriptors have been applied mainly to product discrimination or grading, rather than to decoding the sensory vocabulary used to judge their appearance.

Recent advances in computer vision and machine learning have introduced effective methodologies for tea-appearance feature recognition in agricultural products. Image-based extraction of shape, color, and texture features, when coupled with powerful classifiers like support vector machine (SVM), random forest (RF), and convolutional neural network (CNN), has notably advanced the automated identification of tea varieties, origin tracing, and grade differentiation. For instance, integrating six morphological features derived from computer vision with a polynomial-kernel LS-SVM classifier, has been shown to provide a feasible and effective strategy for rapid and reliable quality grading of Congou black tea, confirming the viability of shape-based features as robust quality identifiers [9]. Similarly, Song et al. achieved near-perfect classification for seven-grade Keemun black tea by fusing near-infrared spectral data with computer vision-derived geometric features and applying a feature-level fusion strategy with softmax-based classification [10]. Li et al. demonstrated that integrating computer vision–based color feature extraction with machine learning classifiers, enables objective and highly accurate evaluation of needle-shaped green tea color quality [11]. Deep-learning detectors have likewise been applied to the appearance-based sorting of machine-picked fresh leaves, which are characterized by small, densely packed and variable in morphology. For example, an enhanced YOLOv12 framework, augmented with modules designed to heighten the response to fine bud features, achieved a precision of 81.2%, a recall of 90.6%, and an mAP@0.5 of 92.7% in recognizing premium buds [12]. In parallel, Huang et al. fused 27 image features (contour-shape, colour and texture) to identify the six grades of Dafo Longjing tea, and further trained three convolutional neural networks to classify four appearance terms, including straightness, as two-level labels with an accuracy up to 99.51% [13]. In the approach, straightness serves merely as a prediction label for the network, and the analysis does not elucidate which specific leaf measurements drive this classification. However, the majority of existing work focuses on global quality assessment rather than on the explicit identification and digital characterization of specific quality traits. This methodological gap highlights the need for interpretable, feature-level frameworks that can bridge sensory terminology and quantitative morphology. To address this gap, this study proposes an interpretable machine learning approach for quantifying the “straightness” morphology of Longjing tea, with three main objectives: (1) extract morphologically meaningful features from tea images and evaluate their effectiveness in discriminating between “straightness” and “non-straightness” samples; (2) develop and compare multiple machine learning classifiers for automated identification; and (3) identify the key features driving the “straight” classification and interpret their alignment with sensory descriptors. By establishing a direct correspondence between sensory vocabulary and objective morphological parameters, this study provides a quantifiable and interpretable framework for standardizing the evaluation of the straightness attribute in Longjing tea, thereby facilitating a paradigm shift from traditional experience-based assessment to data-driven quality control.

## 2. Materials and Methods

### 2.1. Sample Preparation

A total of 37 reference materials for Longjing tea were collected from 18 designated tea-producing counties/districts across 3 major production regions to establish a sample pool, which was summarized in Table 1. One-bud-one-leaf fresh shoots, which were plucked in spring, then were processed following DB33/T 239 [14] and T/ZJTSS 040 [15] through a sequential routine of spreading, first panning, cooling and moisture redistribution, second panning, final panning, sieving, air classification, color sorting, and impurity removal to produce tea samples. The samples were further screened to obtain the final reference materials, which fully complied with the requirements of the national standard for the product of geographical indication—Longjing tea (GB/T 18650) [16]. The prepared materials were then sealed in food aluminum foil bags and stored at −20 °C for subsequent analysis.

### 2.2. Image Acquisition

The 37 reference materials were selected to ensure geographic and processing coverage. For each sample, 18–23 images of individual leaves were acquired, making the individual leaf the unit of observation. Images were captured using a Canon EOS R50 camera (Canon Inc., Tokyo, Japan) equipped with an RF-S 18–45 mm f/4.5–6.3 IS STM lens (aperture f/6.3, shutter speed 1/160 s, ISO 100), mounted at a fixed working distance of 40 cm with a fixed focal length set to 22 mm. Illumination was provided by two white LED panels directed at the sample plane, and the leaves were placed on a white background. Images were acquired at the sensor’s full resolution (6000 × 4000 pixels) and cropped to 450 × 700-pixel tiles without resampling. Because the working distance and focal length remained constant throughout acquisition, the same nominal object-plane scale applies to all 723 images. For an APS-C sensor (22.3 × 14.9 mm; pixel pitch 3.72 µm), a focal length of 22 mm at a working distance of 400 mm gives a magnification of *f*/(*d* − *f*) = 0.058, and consequently an object-plane scale of 0.064 mm per pixel (15.7 pixels per mm). Residual lens distortion may introduce a variation of a few percent across the field of view. Each image contains a single leaf, and each leaf was measured and rated individually. Following the requirements of the national standard Methodology for Tea Sensory Evaluation (GB/T 23776) [17], a panel of five professional panelists (three females and two males) was recruited to evaluate the straightness characteristic of the collected tea leaves. The “straightness” attribute was evaluated based on the textual description in the national standard Tea Vocabulary for Sensory Evaluation (GB/T 14487). Meanwhile, all panelists were recruited from Longjing tea-producing regions and possessed precise recognition of Longjing tea characteristics. Experts in the panel conduct independent evaluations. Following the sensory evaluation criteria, each individual tea leaf was independently assessed by the expert as either “straightness” or “non-straightness”. Using a majority voting rule, a leaf was classified into a specific category if it received three or more votes (≥3). Among the 723 leaves, 532 (73.58%) reached full consensus (5/5), 132 (18.26%) and 59 (8.16%) obtained consensus from four (4/5) and three (3/5) panelists, respectively. Ultimately, all the tea leaves were divided into two categories, namely “straightness” group (*n* = 520) and “non-straightness” group (*n* = 203).

### 2.3. Image Preprocessing

The original image preprocessing was performed using the OpenCV library. The original color images were first converted to grayscale, and the resulting grayscale images were subsequently denoised using a Gaussian blur with a 9 × 9 kernel and a standard deviation of 9 to facilitate skeletonization and clear-structural extraction. Binarization was then carried out via Otsu’s adaptive thresholding method to extract contours, from which the largest contour by area was selected as the region of interest for subsequent feature analysis.

### 2.4. Morphological Feature Extraction

**Figure 1:**
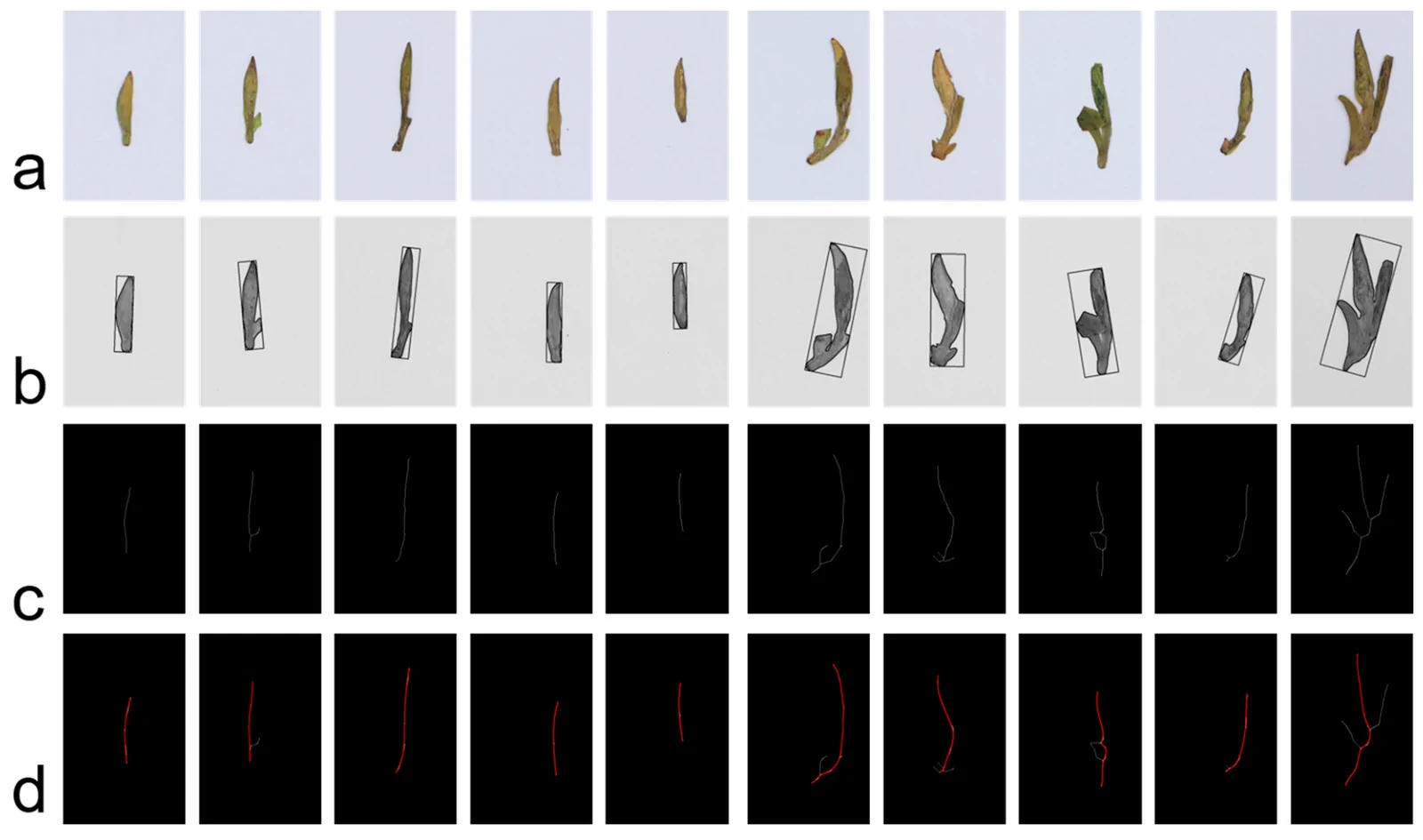
Schematic of the Image Processing and Morphological Feature Extraction Pipeline: (**a**) original tea sample image; (**b**) contour extraction and minimum bounding rectangle; (**c**) skeletonized image; (**d**) main axis extraction and smoothing.

The minimum bounding rectangle was determined from the extracted contour of each tea leaf. Using the contour area (S), perimeter (P), the lengths of the major axis (W) and minor axis (H) of the bounding rectangle, six morphological descriptors were derived, including circularity (E), diameter (D), compactness (J), rectangularity (R), diagonal length (Dia), and slenderness (T), following the definitions reported in previous work [18]. To further characterize the skeletal morphology, the largest contour was rendered onto a blank canvas and re-binarized to ensure a standardized binary format. Skeletonization was then performed using the Zhang–Suen thinning algorithm [19]. Preliminary tests confirmed that the pre-determined Gaussian blur parameters (9 × 9 kernel, σ = 9) effectively suppressed minor surface textures, yielding a cleaner and more concise skeleton. From this skeleton, the coordinates of all pixel points were extracted. Given the near-vertical leaf orientation during image acquisition, the points with the minimum and maximum y-coordinates were designated as the two endpoints of the primary axis. An undirected graph was constructed based on the 8-neighborhood connectivity of skeleton pixels, and Dijkstra’s algorithm was applied to compute the shortest path between the two endpoints, which served as the primary axis [20]. The primary axis was further processed via B-spline interpolation and smoothing to remove serrations and noise from skeleton pixels, producing a smoother and more continuous primary axis curve (Figure 1). Three key features are extracted from this skeletal primary axis, namely average curvature (ac), standard deviation (sd), and length ratio (lr). The formal definitions and computational formulas for all extracted features are summarized in Table 2.

### 2.5. Machine Learning Models Development

Three machine learning algorithms random forest (RF), extreme gradient boosting (XGBoost), and support vector machine (SVM) were employed to classify the “straightness” characteristic of Longjing tea leaves based on the extracted morphological features. All selected features were utilized as input variables, with the binary class label (“straight” = 1, “not straight” = 0) serving as the output, to construct the classification dataset. Prior to model training, all morphological features were standardized using Z-score normalization to eliminate scale effects and ensure comparability across variables. To account for the moderate class imbalance in the dataset (“straight”:“not straight” ≈ 2.56:1), stratified sampling was applied to preserve the original class distribution in both training and testing sets. To mitigate the influence of random variability in data partitioning, each experiment was independently repeated under 10 different random seeds. In each run, the dataset was randomly divided into training and testing sets at an 8:2 ratio. The final model performance was reported as the mean ± standard deviation (Mean ± SD) over the 10 repeated trials. To fully exploit the potential performance of each model, hyperparameter optimization was performed for every experimental run using 10-fold stratified cross-validation combined with GridSearchCV. The optimization objective was to maximize the Area Under the Receiver Operating Characteristic Curve (AUC). Based on preliminary experiments, the hyperparameter space was constrained to conservative ranges to reduce the risk of overfitting. Specifically, the hyperparameter tuning ranges for RF included: the number of decision trees (200, 300), maximum depth (3, 4, 5), minimum samples per leaf (8, 12), and maximum features (‘sqrt’). For XGBoost, the tuned parameters encompassed learning rate (0.05, 0.1), number of trees (100, 150), maximum depth (2, 3), L2 regularization parameter (10, 50, 100), minimum child weight (5, 8), and subsampling ratio (0.7, 0.8). SVM with the RBF kernel was optimized over penalty parameter C (0.1, 1, 10) and gamma (‘scale’, ‘auto’).

### 2.6. SHAP Analysis

SHapley Additive exPlanations (SHAP), a game-theoretic framework that quantifies the marginal contribution of each feature to the prediction for each sample, thereby enabling both global and local interpretability [21,22]. For RF and XGBoost, the TreeExplainer was utilized to compute SHAP values efficiently. For the SVM, which does not have a native tree structure, the model-agnostic KernelExplainer was adopted to approximate SHAP values. All SHAP values were computed for the positive class (straight tea leaves). SHAP values were calculated on the test set for each run. The mean absolute SHAP value of each feature was averaged across the 10 runs and reported as mean ± standard deviation. The summary plots were generated by concatenating SHAP values from all test samples across runs.

### 2.7. Statistical Analysis

Statistical analysis was performed using Python (version 3.9.5). Image processing was carried out with OpenCV (version 4.13.0). the classifiers and hyperparameter search were implemented with scikit-learn (version 1.6.1) and XGBoost (version 2.1.4), and model interpretation with SHAP (version 0.47.2). Numerical operations used NumPy (version 2.0.2) and pandas (version 2.3.3). The violin plots were generated using Seaborn (version 0.13.2) and Matplotlib (version 3.9.4) in Python. The normality of the data was assessed using the Shapiro–Wilk test from the SciPy library (version 1.13.1). Differences between the two groups were evaluated using the non-parametric Mann–Whitney U test (mannwhitneyu function in SciPy, version 1.13.1), with the level of significance set at *p* < 0.05. To quantify the magnitude of the observed differences, Cliff’s delta (δ) was computed using a custom Python implementation based on the formulation described by Cliff [23]. Both the corrected resampled *t*-test [24] and the Holm procedure were implemented in Python with SciPy (version 1.13.1). Spearman’s rank correlation coefficients among the morphological features were calculated using the corr method of the pandas library with method = ‘spearman’. The resulting correlation matrix was visualized as a half-matrix heatmap using Python and the Matplotlib library.

## 3. Results and Discussion

### 3.1. Univariate Statistical Analysis of Morphological Features

**Figure 2:**
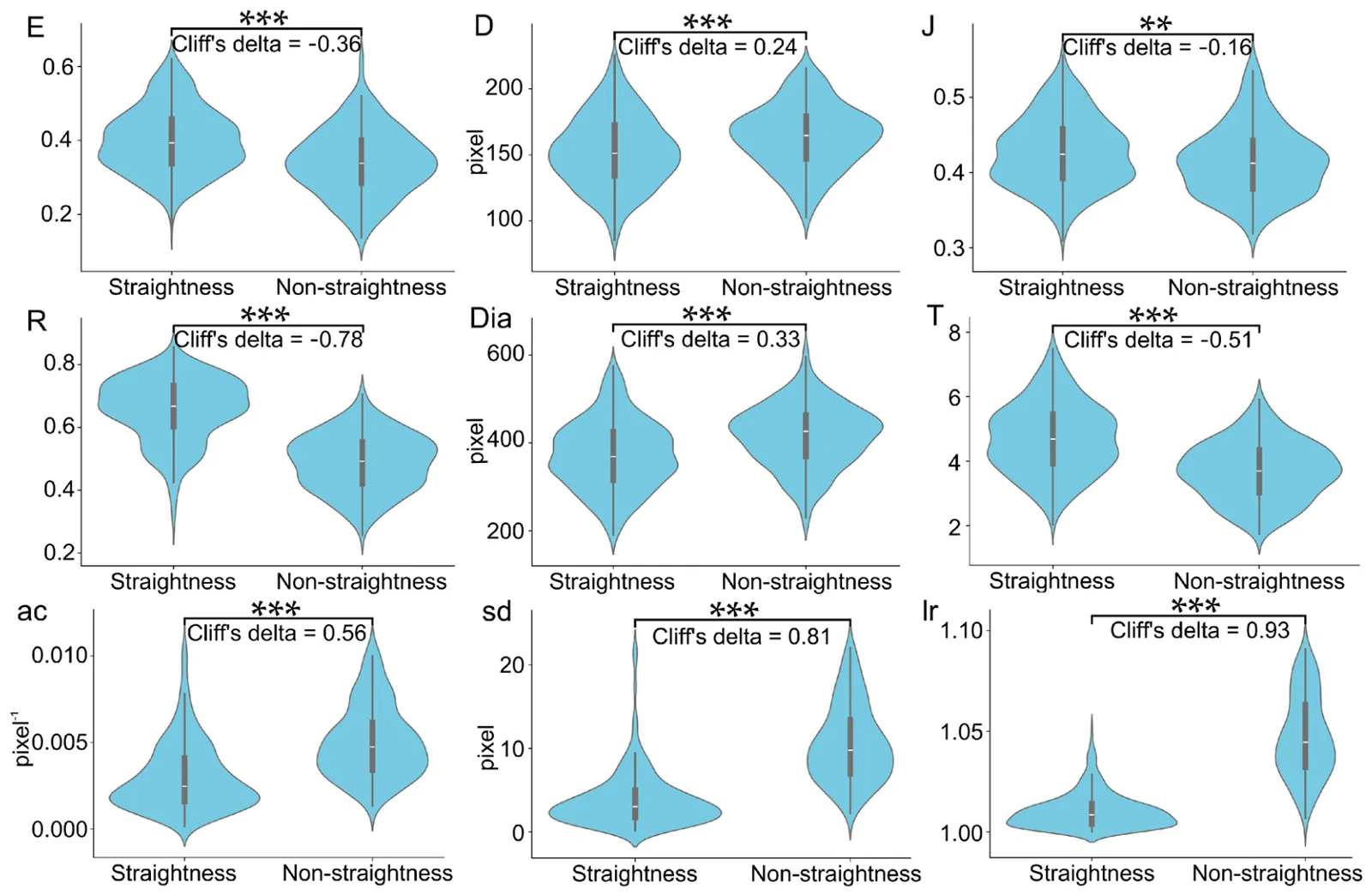
Violin plots of morphological features. Asterisks indicate the significance level of the Mann–Whitney U test (** *p* < 0.01, *** *p* < 0.001).

The violin plots of the nine morphological features provide a visual representation of their respective distributions across the “straightness” and “non-straightness” categories (Figure 2). Given that the most features deviated from normality (Shapiro–Wilk test, *p* < 0.05), between-group comparisons were conducted using the Mann–Whitney U test, with effect size quantified by Cliff’s delta (δ). This metric ranges from −1 to 1, where δ > 0 indicates that a randomly selected sample from the “non-straightness” group tends to have a higher feature value than one from the “straightness” group, and δ < 0 reflects the opposite tendency. The magnitude of |δ| reflects the degree of distributional separation between the two groups, with values approaching 1 indicating progressively large differences. As shown in Figure 2, the Mann–Whitney U test revealed that feature J exhibited a significant difference between the two groups (*p* < 0.01), while the remaining eight features showed highly significant differences (*p* < 0.001). However, considerable variation was observed in the effect sizes (δ) among these eight features. The magnitude of δ was closely associated with the degree of distribution overlap visualized in the violin plots: higher δ values corresponded to lower overlap, and conversely, lower δ values reflected greater overlap between the two groups.

The shape of the main axis was quantified by three morphological features extracted from the tea skeleton, namely ac, sd and lr. Among these, lr (δ = 0.93) and sd (δ = 0.81) exhibited the strongest discriminatory power for straightness classification. Since lr precisely measures the excess of path length over endpoint distance [25], its leading position in feature importance is expected. These two descriptors capture distinct geometric properties of the same axis: lr is the ratio of the path length to the distance between the two endpoints, such that every increment of bending along the axis is accumulated into a single value. In contrast, sd represents the standard deviation of the perpendicular distances from the axis points to the least-squares line, thereby reflecting the amplitude of the deviation rather than its accumulation. Although both features shifted towards higher values in the non-straightness group, their violin plots (Figure 2) revealed distinct patterns of separation. For lr, the two distributions were narrow with minimal overlap, with each group occupying a separate region along the axis. By contrast, the violins for sd overlapped considerably along much of their length: the straightness distribution was concentrated at the lower end of the scale but retained a long, thin upper tail extending well into the upper half of the non-straightness distribution. This indicates that a minority of leaves classified as straight deviated from the fitted line by an amount comparable to that of non-straight leaves. The feature ac (δ = 0.56) quantifies the average local curvature along the primary axis. Although its discriminatory power is inferior to that of lr and sd, ac is particularly sensitive to subtle local fluctuations in curvature that may be overlooked by the other two metrics. While ac alone is insufficient to determine tea leaf straightness, it serves as a valuable complementary feature to lr and sd.

The values of morphological features R, T and E were significantly higher in the straightness group than in the non-straightness group. Both R and T, derived from the minimum bounding rectangle, reflect the geometric relationship between the tea leaf contour and its bounding box. The feature R (δ = −0.78) quantifies the efficiency with which the leaf contour occupies the bounding rectangle; it yielded significantly higher values in the straightness group because straight leaves have more compact contours that better fill the rectangle, whereas the non-straightness group exhibited lower values because curvature enlarges the rectangular area without a proportional increase in leaf area. Similarly, feature T (δ = −0.51), which represented the aspect ratio of the minimum bounding rectangle, reflected the overall slenderness of the leaf. Higher T values were observed in the straightness group, attributable to a longer primary axis and narrower width. In contrast, the non-straightness group exhibited lower T values, as curvature reduces the projected length of the primary axis along the major axis of the bounding rectangle while simultaneously increasing its width. Although these two features rely on the dimensions of the bounding rectangle and contribute effectively to discriminating between straightness and non-straightness leaves, they differ in their sensitivity: feature R is more responsive to overall compactness of the contour, whereas feature T primarily captures changes in the length-to-width ratio induced by curvature. Feature E exhibited a relatively weaker discriminatory capability, with a Cliff’s δ of −0.36. In theory, similar to R, feature E should be larger in straightness group, as curvature increases the contour perimeter (P) for a given area (A), and a larger denominator (P^2^) leads to a lower E value. However, the observed discriminative power of E was substantially lower than that of R, with considerable overlap between the distributions of the two groups. This discrepancy may be attributed to the high sensitivity of the perimeter P to minor irregularities and fine serrations along the leaf margin. Even samples classified as “straightness” group frequently exhibited non-smooth edges, which artificially increased P, consequently lowering the E value and diminishing its discriminative capability relative to the rectangularity measure R.

Features D, Dia, and J are related to the absolute dimensions and their derived metrics of tea leaves. The positive effect sizes observed for D (δ = 0.24) and Dia (δ = 0.33) indicated that non-straightness samples may have larger overall dimensions than straightness samples. In contrast, feature J exhibited the smallest effect size (δ = −0.16), indicating limited discriminative capability. Defined as the ratio of the equivalent diameter (D) to the width of the minimum bounding rectangle (W), J is subject to an inherent positive correlation between its numerator and denominator, both of which scale with overall leaf size. This autocorrelation substantially attenuated J’s sensitivity to leaf curvature, resulting in its weakest discriminatory capability and the lowest statistical significance between the two groups. Therefore, feature J was excluded from subsequent classification model development, and the remaining eight features were retained.

### 3.2. Models Performance Comparison

To evaluate the discriminative performance of the selected morphological features in classifying tea leaf straightness, we employed three mainstream machine learning models, including Random Forest (RF), Extreme Gradient Boosting (XGBoost), and Support Vector Machine (SVM). Seven metrics were employed to evaluate their classification performance. As straightness was the majority class and was designated as the positive class, accuracy, recall, precision, and F1-score primarily characterize majority/positive-class performance. Specifically, accuracy measures the overall proportion of correctly classified leaves; recall quantifies the proportion of actual straight leaves that were correctly identified; precision represents the proportion of leaves predicted as straightness that were true positives; and the F1-score is the harmonic mean of precision and recall, emphasizing the positive-class performance [26]. Given the class imbalance, these four metrics alone are insufficient to adequately reflect the model’s ability to recognize the minority class. Specificity, defined as the proportion of correctly identified true negatives, directly reflects the recall of the minority negative class. In addition, the Matthews Correlation Coefficient (MCC) provides a comprehensive evaluation based on the confusion matrix [27], whereas ROC-AUC is relatively insensitive to class distribution [28]. Therefore, MCC and ROC-AUC were therefore selected as primary summary metrics, with specificity reported to explicitly quantify minority-class recognition. To ensure a rigorous and unbiased evaluation of classification performance and generalizability, we conducted repeated experiments with 10 different random seeds. For each pair of models and each evaluation metric, the Shapiro–Wilk test was applied to assess the normality of the paired differences across the 10 trials. The results indicated that the paired differences did not deviate significantly from normality. Because the ten splits reuse the same 723 samples, the training sets of different runs overlapped. We therefore applied the corrected resampled *t*-test to adjust the variance estimate of the paired differences, thereby accounting for the dependence induced by this overlap. The resulting p-values were adjusted for all 21 comparisons using the Holm procedure to control the family-wise error rate.

**Figure 3:**
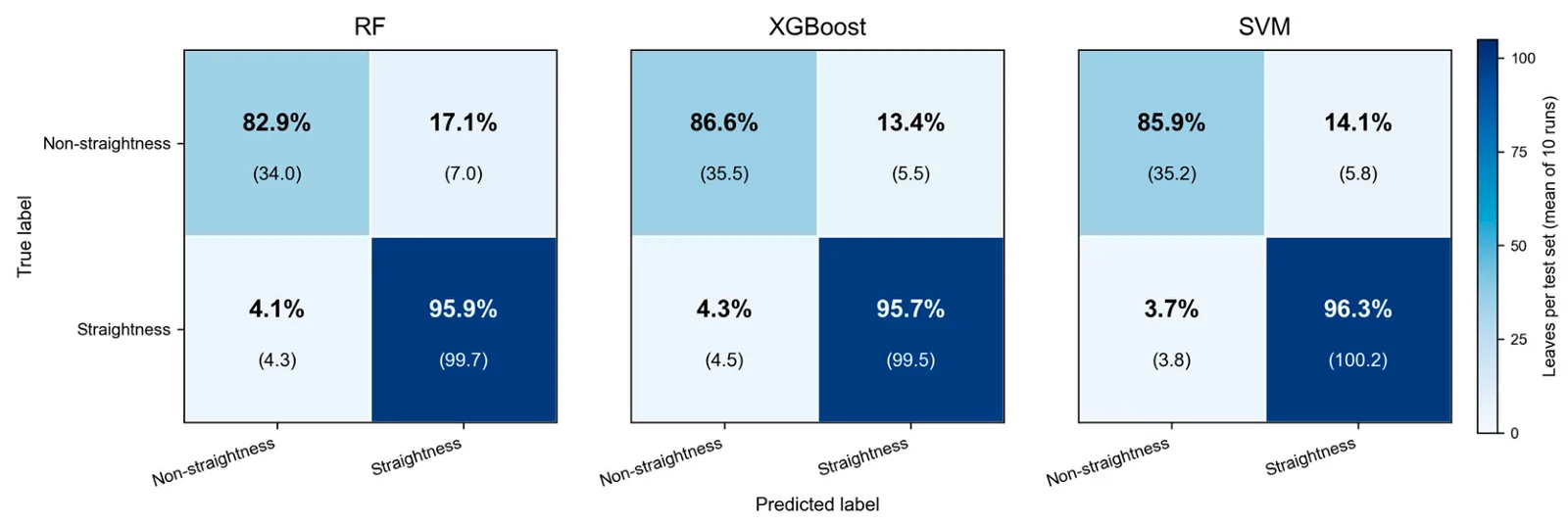
Mean confusion matrices of the three models over the ten repeated runs. Percentages are normalised by row (true class); values in parentheses give the mean number of leaves per test set (145 leaves per run, 1450 in total).

A comprehensive comparison of the model performance across multiple metrics is summarized in Table 3. The primary summary metrics indicated strong overall discrimination (mean ROC-AUC = 0.978). While SVM and XGBoost achieved comparable MCC values, both slightly higher than that of RF, no pairwise difference was statistically significant after Holm correction. For the majority straightness class, test accuracy ranged from 0.922 (RF) to 0.934 (SVM), recall from 0.957 to 0.963, and precision from 0.935 to 0.948, while the mean F1-scores of the three models differed by less than 0.01. Specificity, which directly quantifies recognition of the minority non-straightness class, was comparable between SVM and XGBoost and slightly higher than that of RF. Consistently, the mean confusion matrices in Figure 3 revealed a distinct asymmetry: 13.4–17.1% of non-straightness leaves were misclassified as straightness, versus only 3.7–4.3% of straightness leaves were misclassified in the opposite direction. Across the ten random seeds, the SD was 0.007–0.021 for accuracy, precision, recall, F1-score, and AUC, but reached 0.038 for MCC (SVM) and 0.061 for specificity (SVM). This indicates that AUC and the majority-class metrics were relatively stable, whereas MCC and minority-class recognition were more variable. A similar pattern was observed for training–test gaps: 0.007–0.023 for accuracy, precision, recall, F1-score, and AUC, versus 0.041–0.055 for MCC and 0.033–0.048 for specificity (Table 4).

The larger SD and training–test gaps of specificity and MCC reflect the size of the minority class and the distinct ways in which each metric combines the two classes. Specificity is computed on approximately 41 non-straightness leaves per test set, compared with approximately 104 straightness leaves for recall. Consequently, an equal absolute change in the number of errors yields an approximately 2.5-fold larger change in specificity than in recall. This amplification applies to partition-to-partition variation in specificity relative to recall; it also contributes to, but does not by itself determine, the larger training–test gap. MCC responds to the error rates of both classes. Because the minority error rate exhibits greater variability, it dominates the variance. As a result, the SD therefore lay between that of specificity and those of the majority-class metrics, while its training–test gap exceeded that of specificity in all three models. AUC and the majority-class metrics exhibited the greatest stability: AUC is threshold-free and ranks all test leaves, whereas accuracy, precision, recall and F1-score are dominated by the majority class.

**Figure 4:**
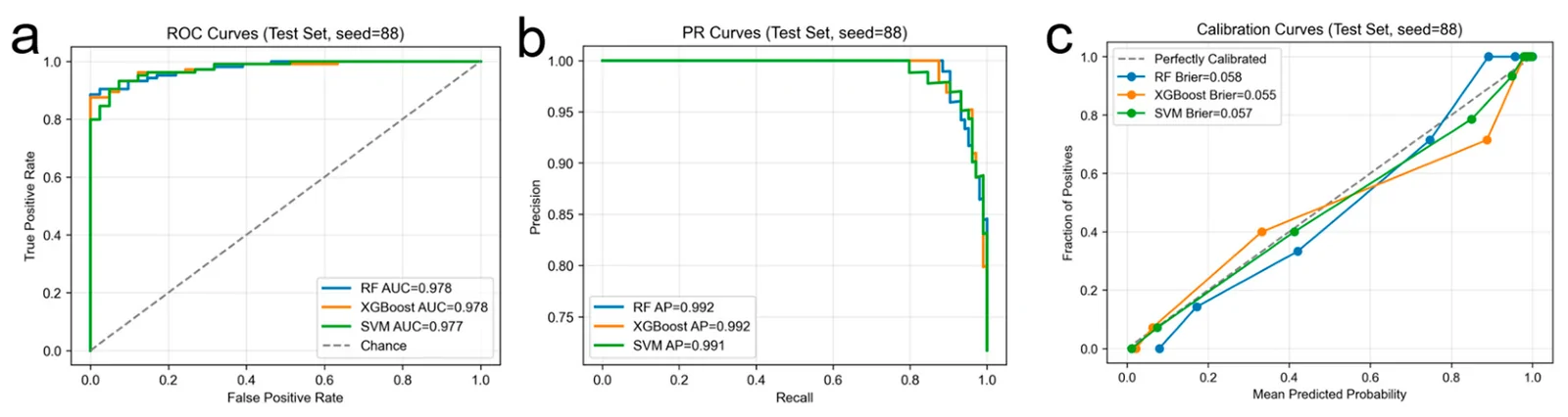
Comparison of ROC, PR, and calibration curves for the three models (RF, XGBoost, and SVM) at seed = 88. (**a**) ROC curves; (**b**) PR curves; (**c**) calibration curves. ROC, receiver operating characteristic; PR, precision–recall; AUC, area under the curve; AP, average precision; Brier, Brier score.

Precision–Recall (PR) curves were subsequently plotted to assess the model’s performance with respect to precision and recall for the positive class. In addition, calibration curves and Brier scores were employed to quantify the accuracy and reliability of the model’s predicted probabilities. Using the representative experiment with random seed 88 as an example, the ROC and PR curves for the three models (Figure 4a,b) showed no substantial separation, indicating comparable performance across thresholds. Calibration curves (Figure 4c) further demonstrated that all three models produced well-calibrated probability estimates, with strong agreement between predicted and observed probabilities. Overall, the three models showed negligible differences in overall ranking ability and probability calibration.

The comparable performance of the three algorithms can be attributed to their distinct underlying mechanisms. Specifically, SVM with an RBF kernel performs implicit margin maximization in a transformed feature space, a formulation generally regarded as robust in small-sample settings [29,30] and suitable under moderate class imbalance [31,32,33]. XGBoost constructs trees iteratively, enabling the capture of nonlinear relationships and feature interactions [34], and its L2 regularization on leaf weights limits overfitting when the sample size is limited [35,36]. RF instead grows trees independently on bootstrap samples and aggregates them by majority voting [37,38]. All three algorithms have been widely applied in tea quality evaluation, including the optimization of rolling parameters [39], the prediction of physicochemical indicators in green tea [40], the estimation of tea polyphenol content [41], and appearance grading [13]. In summary, RF, XGBoost, and SVM all demonstrated strong classification performance and reliable probability calibration for the automated evaluation of tea leaf straightness. Although slight differences were observed in threshold-dependent metrics, the three models exhibited comparable overall ranking capability and stability in the present study. Therefore, subsequent SHAP analysis was performed using the predictive outputs of all three models to provide a comprehensive interpretation and further enhance the reliability of the results.

### 3.3. SHAP-Based Interpretation of Feature Contributions

**Figure 5:**
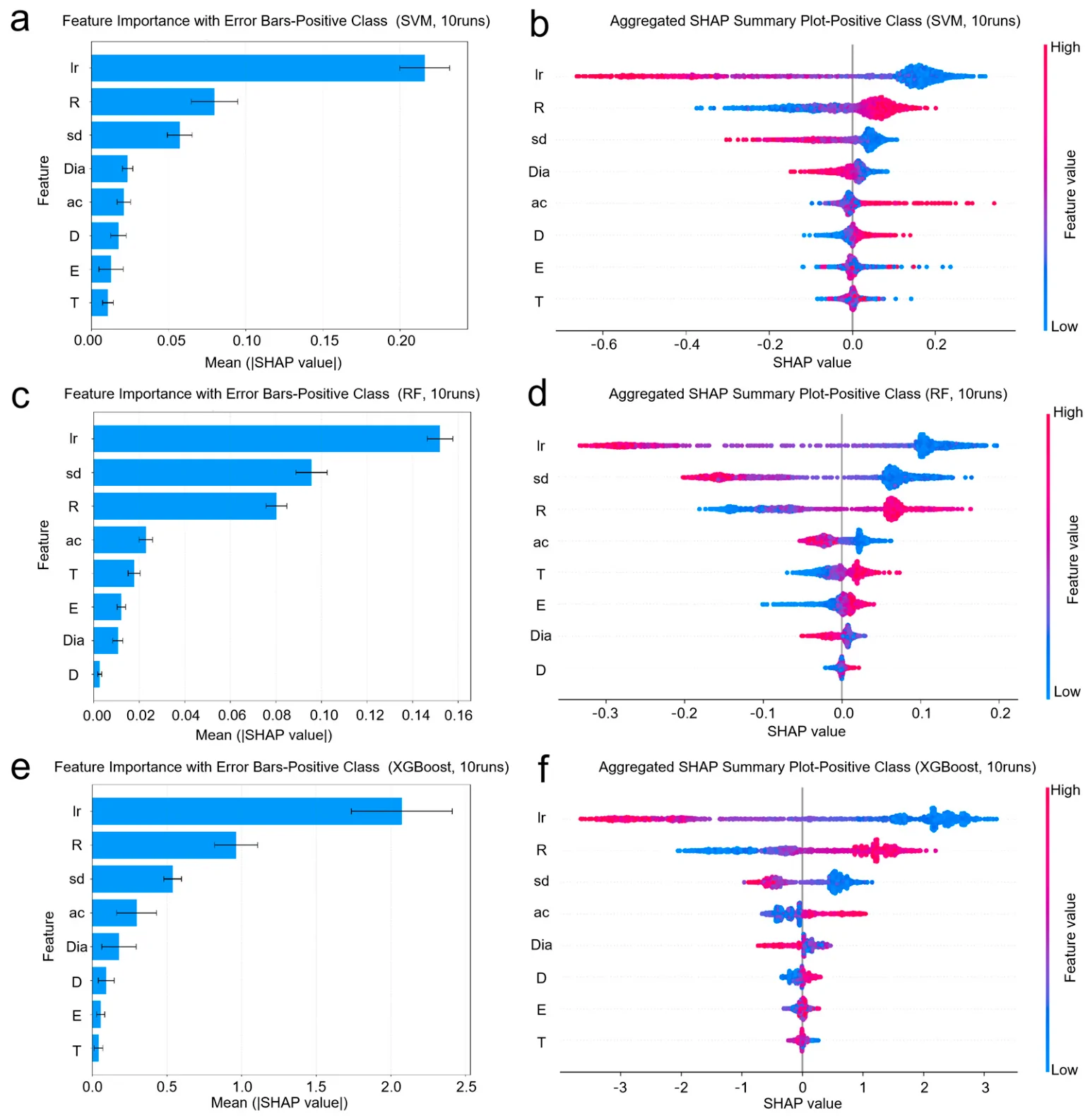
Aggregated SHAP feature importance for three models across ten independent random seeds. (**a**,**c**,**e**) Mean absolute SHAP value with SD over the ten runs; (**b**,**d**,**f**) corresponding summary plots. The *x*-axis of (**e**) covers a different range from that of (**a**,**c**), because SHAP values carry the unit of the model output: the predicted probability in RF and SVM, but log-odds in XGBoost. The bars in (**e**) are therefore numerically larger without indicating a larger effect: bar lengths are comparable within a panel, and across panels only the order of the features is comparable.

To thoroughly elucidate the contribution of each morphological feature to the model’s predictions, this study employed the SHAP (SHapley Additive exPlanations) value to interpret feature importance across RF, XGBoost, and SVM models [21,22]. SHAP summary plots and bar charts of feature importance with error bars were generated to visually illustrate the influence of each feature on the model’s output. Based on the SHAP results from all three models [42,43], the contribution levels of individual features in distinguishing straightness from non-straightness were compared, enabling the identification of the most critical morphological descriptors. The mean and standard deviation of the average absolute SHAP values for each feature were calculated across ten independent experiments with different random seeds for all three models, as shown in Figure 5a,c,e. Among the three models, lr consistently emerged as the most important feature, with SHAP values significantly higher than those of all other features. This dominance is expected, given that lr is defined as the ratio of the axis length to the distance between its endpoints. Its value equals 1 for a perfectly straight axis and increases as the axis bends, meaning the descriptor inherently encodes the morphological distinction separating the two classes. The features sd and R consistently held the second and third ranks across all models, although their relative ordering varied slightly between models (RF: lr > sd > R; XGBoost and SVM: lr > R > sd). The remaining features (ac, Dia, T, E, D) exhibited markedly lower mean absolute SHAP values with only minor inter-model variations, placing them at the periphery of model decision-making and indicating their relatively limited overall contribution. The SHAP summary plots (Figure 5b,d,f) further illustrated the directional influence of each feature on model predictions. Each point in the plot corresponded a test sample, with color indicating feature value (from blue for low to red for high), and the horizontal position reflecting the feature’s contribution to the prediction. Positive SHAP values indicated that the feature pushes the model output toward the positive class (straightness), whereas negative values drove it toward the negative class. Across all three models, lr exhibited a clear negative correlation with SHAP values: samples with low lr values were predominantly located on the positive SHAP side, strongly contributing to straightness classification. Similarly, feature sd also displayed a negative correlation. In contrast, feature R exhibited a positive correlation, with high-R samples primarily distributed on the positive SHAP side. For features such as Dia and those with lower importance rankings, the color stratification was less distinct, suggesting that although these features provided some predictive information, their contributions were relatively limited.

### 3.4. Correlation Analysis

**Figure 6:**
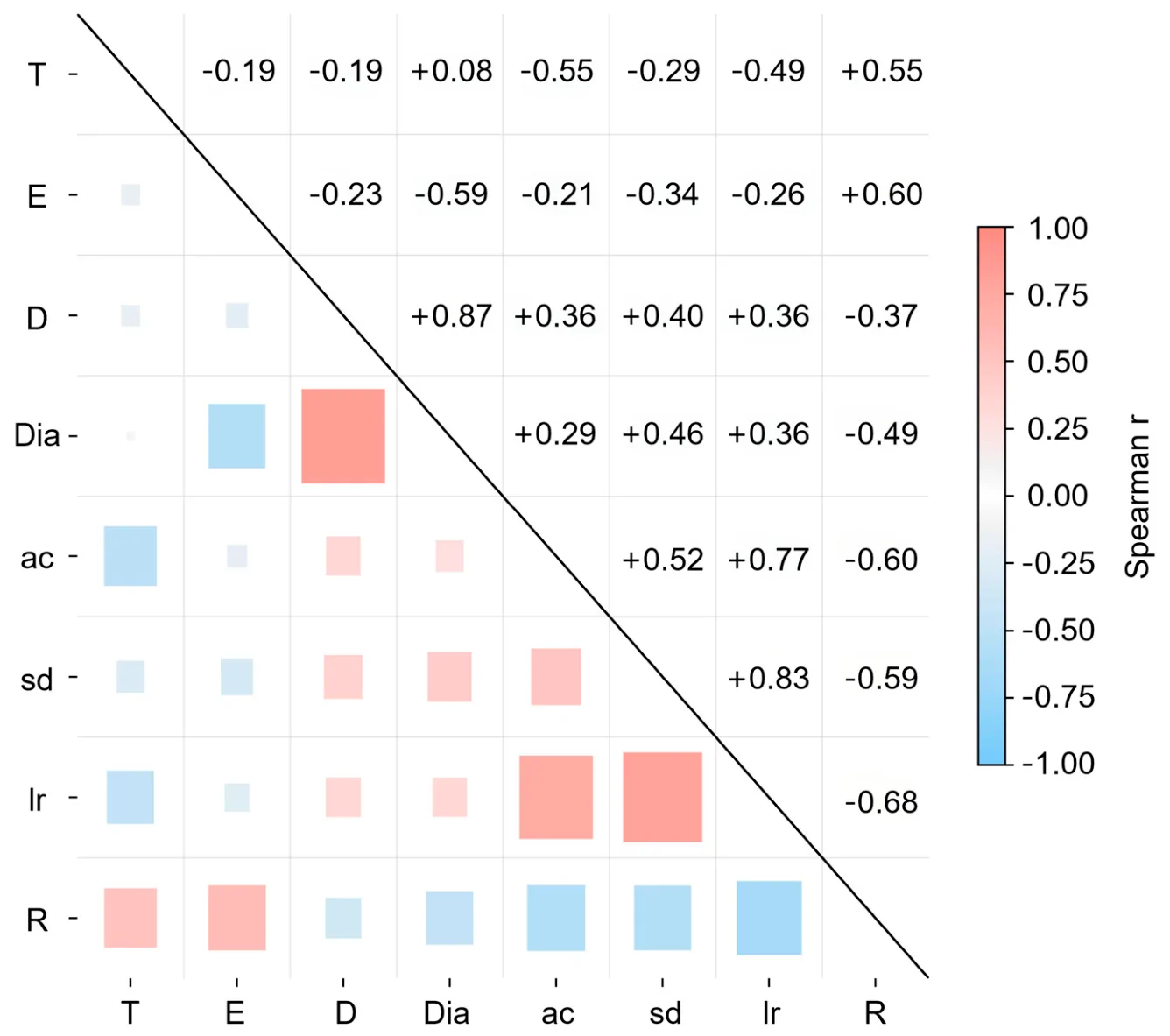
Correlation heatmap of the morphological features.

To further elucidate the determinants of feature importance rankings, we performed an integrative analysis combining SHAP values, univariate statistical effect sizes (δ), and inter-feature correlations (Figure 6). The feature importance rankings derived from SHAP analysis were highly consistent with the univariate discriminative power of the corresponding features. Specifically, features such as lr, R, and sd consistently ranked among the top three features across all three models. Accordingly, Cliff’s δ values (Figure 2) indicated that lr (0.93), sd (0.81), and R (−0.78) exhibited exceptionally strong univariate separability between the two classes. Moreover, the results of Cliff’s δ aligned well with the directional influence observed in the SHAP summary plots: low lr and low sd values drove predictions toward the positive class (straightness), while high R values contributed similarly. This concordance confirmed the consistency between univariate analysis and the multivariate model interpretations.

As shown in Figure 6, correlation analysis further elucidated the interplay between information redundancy and complementarity among these features. The strong positive correlation between lr and sd (r = 0.83) indicated substantial redundancy, whereas the negative correlations of R with lr (r = −0.68) and with sd (r = −0.59) suggested that R carried partially independent information related to leaf compactness. This pattern is consistent with their definitions: lr accumulates bending along the primary axis while sd quantifies the amplitude of deviation from a straight configuration, so that both describe the same axis geometry at different scales, whereas R reflects how fully the two-dimensional contour fills its bounding rectangle, an area-based property that the axis-based descriptors cannot represent. Although sd exhibited strong univariate discriminative power (δ = 0.81), its unique contribution was smaller than that of R, because much of its ordering is shared with lr. Because lr is strongly correlated with sd (r = 0.83) and ac (r = 0.77), the credit that the models assign to each of the three is not unique, so only their joint ranking is read from the SHAP values. A comparison was conducted between a logistic regression using lr alone and its nested extensions (sd and R) under identical conditions (the same 723 samples and standardized features). The results demonstrated that the multivariate model achieved superior classification performance, and also showed that both descriptors carried additional information beyond lr (Table S1).

Beyond the three primary features, all remaining features showed minimal contribution in the SHAP analysis, and the five together accounted for 16–20% of the total. This limited contribution can be attributed to two factors, information redundancy with the core features and inherently weak individual discriminative power. The first category includes features that were highly correlated with the primary features, notably ac and T. Although ac exhibited moderate univariate discriminative ability (δ = 0.56) compared to lr, R, and sd, its strong correlation with lr (r = 0.77) and its characterization of local axis fluctuations led to a reduced contribution. Similarly, feature T, despite a moderate-to-large effect size (δ = −0.51), showed correlations of approximately 0.5 with both lr and R, indicating considerable overlap with the core features and resulting in lower SHAP ranking. The second category encompasses features with intrinsically limited discriminative power and high inter-feature redundancy, specifically Dia, D, and E. These three features were strongly interrelated (Dia vs. D: r = 0.87; Dia vs. E: r = −0.59), forming a closely associated cluster. More importantly, their Cliff’s Delta effect sizes were uniformly small (absolute values ranging from 0.24 to 0.36), indicating weak univariate discriminative capacity. The combination of low discriminability and high redundancy rendered these three features essentially negligible in model decision-making, accounting for their extremely low SHAP contributions. In summary, features with high discriminative power (lr, R, sd) were identified as core features; those with moderate discriminative ability but high redundancy with the core features (ac and T) were downweighted; and features with low discriminative power and internal redundancy (Dia, D and E) contributed negligibly. Notably, the consistent identification of lr, R, and sd as core features across three fundamentally different modeling algorithms suggested that these features are not artifacts of model-specific inductive biases.

## 4. Conclusions

This study developed an interpretable machine learning framework for the automated evaluation of leaf straightness, a key sensory attribute in Longjing tea quality evaluation. From 723 images, nine morphological features were extracted and used to train three classifiers, including Random Forest (RF), Extreme Gradient Boosting (XGBoost), and Support Vector Machine (SVM). SHAP-based interpretability analysis identified three core morphological features, namely axis length ratio (lr), standard deviation of the distance from the primary axis point to the fitted line (sd), and rectangularity (R). The low lr, low sd, and high R values were found to be strongly associated with the straightness attribute. These features exhibited strong univariate discriminative power and cross-model feature importance rankings. Correlation analysis confirmed that lr and sd were substantially correlated (r = 0.83), while R provided complementary information related to leaf compactness. The proposed system successfully translates a traditionally subjective sensory descriptor into quantifiable and interpretable digital indicators. This work not only provides a foundation for automating tea quality evaluation, but also offers a methodological framework for digitizing sensory morphology in other agricultural products, supporting the broader movement toward data-driven standardization in food quality assessment. Although the reference library comprehensively encompassed 18 tea-producing counties/districts across 3 production regions, the leaf-level data split indicated that the reported performance reflects new leaves from represented samples rather than entirely unseen ones. In addition, the spatial scale of the images was nominal (pixel-based) rather than physically calibrated; consequently, length-based descriptors (D, Dia, sd, ac) were comparable only under a fixed acquisition geometry. Future work will therefore integrate sample-grouped validation, instrument calibration and an inter-laboratory reproducibility trial with an expanded sample library, to translate these criteria into a standardized measurement protocol.

## Figures and Tables

**Table 1:** Sample information.

Region	Counties/Districts	Number of Samples
Xihu producing areas	Xihu District	3
Qiantang producing areas	Xiaoshan District	3
	Binjiang District	1
	Yuhang District	2
	Fuyang District	3
	Linan District	1
	Tonglu County	2
	Jiande County	3
	Chunan County	3
Yuezhou producing areas	Shaoxing City	1
	Yuecheng District	1
	Xinchang County	3
	Shengzhou County	3
	Zhuji County	2
	Shangyu District	1
	Panan County	2
	Dongyang County	2
	Tiantai County	1

**Table 2:** Feature description.

Feature	Definition	Computational Formulas
E	Measure of the similarity between the contour shape and a perfect circle	$E=\frac{4\mathrm{\pi S}}{P^{2}}$
D	Diameter of a circle with the same area as the tea contour	$D=2\sqrt{\frac{S}{\pi }}$
J	Ratio of D to the width of the minimum bounding rectangle of the tea leaf	$J=\frac{D}{W}$
R	Ratio of tea leaf area to the area of the minimum bounding rectangle	$R=\frac{S}{W\times H}$
Dia	Diagonal length of the minimum bounding rectangle of the tea leaf	$\mathrm{Dia}=\sqrt{W^{2}+H^{2}}$
T	Ratio of the long side to the short side of the minimum bounding rectangle	$T=\frac{W}{H}$
ac	Mean curvature of the primary axis	$\mathrm{ac}=\frac{1}{N}\sum_{i=1}^{N}k(t_{i})$
sd	Standard deviation of the perpendicular distances from all points on the primary axis to the fitted line	$\mathrm{sd}=\sqrt{\frac{1}{N}\sum_{i=1}^{N}(d_{i}-\overset{-}{d}{)}^{2}}$
lr	Ratio of the total length of the primary axis to the straight-line distance between its endpoints	$\mathrm{lr}=\frac{L_{1}}{L_{2}}$

Note: *S* denotes the tea leaf area, defined as the number of pixels within the largest contour of the tea leaf; *P* represents the perimeter, defined as the total number of pixels along the largest contour; *W* and *H* are the length and width of the minimum bounding rectangle, respectively; *k*(*t_i_*) is the curvature at the *i*-th point on the primary axis, computed as the reciprocal of the local radius of curvature and expressed in pixel^−1^; ac is the mean of *k*(*t_i_*) along the primary axis. The least squares method is used to fit a straight line to all points on the smoothed primary axis, where *d_i_* is the perpendicular distance from the *i*-th point on the primary axis to the fitted line, and $\overset{-}{d}$ is the mean perpendicular distance from all points on the primary axis to the best-fit line; *L*_1_ is the total length of the primary axis, and *L*_2_ is the straight-line distance between the two endpoints of the primary axis. lr = *L*_1_/*L*_2_ is dimensionless and ≥1, equalling 1 only for a perfectly straight axis.

**Table 3:** Overall performance of the three machine learning models.

Models		RF	XGBoost	SVM
Train dataset	Accuracy	0.945 ± 0.005	0.948 ± 0.007	0.954 ± 0.005
	Precision	0.953 ± 0.005	0.961 ± 0.007	0.959 ± 0.007
	Recall	0.971 ± 0.005	0.968 ± 0.004	0.978 ± 0.004
	F1-Score	0.962 ± 0.003	0.964 ± 0.005	0.968 ± 0.004
	AUC	0.988 ± 0.002	0.988 ± 0.003	0.985 ± 0.003
	MCC	0.861 ± 0.012	0.872 ± 0.018	0.885 ± 0.014
	Specificity	0.877 ± 0.014	0.899 ± 0.018	0.894 ± 0.019
Test dataset	Accuracy	0.922 ± 0.009	0.931 ± 0.012	0.934 ± 0.015
	Precision	0.935 ± 0.013	0.948 ± 0.017	0.946 ± 0.021
	Recall	0.959 ± 0.021	0.957 ± 0.019	0.963 ± 0.011
	F1-Score	0.946 ± 0.007	0.952 ± 0.009	0.954 ± 0.010
	AUC	0.978 ± 0.008	0.978 ± 0.008	0.978 ± 0.007
	MCC	0.807 ± 0.023	0.830 ± 0.031	0.835 ± 0.038
	Specificity	0.829 ± 0.040	0.866 ± 0.048	0.859 ± 0.061

Note: Values are the mean ± SD of ten repeated runs. Pairwise differences between the models were assessed with the Nadeau–Bengio corrected resampled *t*-test and adjusted with the Holm procedure across all 21 comparisons (three model pairs × seven metrics); no difference was significant (adjusted *p* > 0.05).

**Table 4:** Gap between the train and test sets for the RF, XGBoost, and SVM models.

Model	Metric	Train	Test	Gap
RF	Accuracy	0.945 ± 0.005	0.922 ± 0.009	0.023
	Precision	0.953 ± 0.005	0.935 ± 0.013	0.018
	Recall	0.971 ± 0.005	0.959 ± 0.021	0.012
	F1-Score	0.962 ± 0.003	0.946 ± 0.007	0.016
	AUC	0.988 ± 0.002	0.978 ± 0.008	0.01
	MCC	0.861 ± 0.012	0.807 ± 0.023	0.055
	Specificity	0.877 ± 0.014	0.829 ± 0.040	0.048
XGBoost	Accuracy	0.948 ± 0.007	0.931 ± 0.012	0.017
	Precision	0.961 ± 0.007	0.948 ± 0.017	0.013
	Recall	0.968 ± 0.004	0.957 ± 0.019	0.011
	F1-Score	0.964 ± 0.005	0.952 ± 0.009	0.012
	AUC	0.988 ± 0.003	0.978 ± 0.008	0.01
	MCC	0.872 ± 0.018	0.830 ± 0.031	0.041
	Specificity	0.899 ± 0.018	0.866 ± 0.048	0.033
SVM	Accuracy	0.954 ± 0.005	0.934 ± 0.015	0.02
	Precision	0.959 ± 0.007	0.946 ± 0.021	0.013
	Recall	0.978 ± 0.004	0.963 ± 0.011	0.015
	F1-Score	0.968 ± 0.004	0.954 ± 0.010	0.014
	AUC	0.985 ± 0.003	0.978 ± 0.007	0.007
	MCC	0.885 ± 0.014	0.835 ± 0.038	0.05
	Specificity	0.894 ± 0.019	0.859 ± 0.061	0.035

Note: Gap = mean value (Train) − mean value (Test), calculated from the corresponding means of the 10 runs. AUC: Area Under the Curve. MCC: Matthews Correlation Coefficient.

## Data Availability

The original contributions presented in this study are included in the article/Supplementary Materials. Further inquiries can be directed to the corresponding author.

## Supplementary Materials

The following supporting information can be downloaded at: https://www.mdpi.com/article/10.3390/foods15213983/s1, Table S1: Nested logistic regression models assessing the incremental value of sd and R over lr.
