# Supplementary material for: Machine Learning and SHAP for Morphological Feature Identification of Longjing Tea Straightness
Source: Foods. 2026 Nov 6;15(21):3983. doi: 10.3390/foods15213983 (PMC13762581; doi:10.3390/foods15213983)
Supplement: Supplementary file 1 [file foods-15-03983-s001.zip › foods-4588642-supplementary.pdf]

Supplementary Materials

Table S1 Nested logistic regression models assessing the incremental value of sd and R over lr.

Model	AIC	Δ AIC	p
lr	317.73	—	—
lr + sd	310.81	−6.92	0.0028
lr + R	259.77	−57.96	9.7×10^{-15}
lr + sd + R	253.15	−64.58	1.3×10^{-15}

Note: AIC is defined as −2 ln L + 2k, where L is the maximized likelihood and k the number of estimated parameters including the intercept; lower values indicate a better trade-off between goodness of fit and model complexity. ΔAIC and p are both calculated relative to the lr-only model for all rows, so that negative values denote an improvement. A reduction in AIC of less than 2 indicates practical equivalence, a reduction of 4–7 indicates that the model with the higher AIC (i.e., the model with fewer descriptors) is considerably less plausible, and a reduction greater than 10 indicates that it is essentially unsupported. p is the p-value of the likelihood-ratio test comparing the model in that row with the lr-only model (df = 1 for models containing one additional descriptor; df = 2 for lr + sd + R).
